# Effects of Aqueous Extracts of Endophyte-Infected Grass *Achnatherum inebrians* on Growth and Development of Pea Aphid *Acyrthosiphon pisum*

**DOI:** 10.3390/insects12100944

**Published:** 2021-10-18

**Authors:** Yaling Ma, Chunjie Li, James F. White

**Affiliations:** 1 State Key Laboratory of Grassland Agro-Ecosystems, Lanzhou University, Lanzhou 730020, China; mayl18@lzu.edu.cn; 2 Key Laboratory of Grassland Livestock Industry Innovation, Ministry of Agriculture and Rural Affairs, Lanzhou University, Lanzhou 730020, China; 3 Engineering Research Center of Grassland Industry, Ministry of Education, Lanzhou University, Lanzhou 730020, China; 4 Gansu Tech Innovation Centre of Western China Grassland Industry, Lanzhou University, Lanzhou 730050, China; 5 Center for Grassland Microbiome, Lanzhou University, Lanzhou 730050, China; 6 College of Pastoral Agriculture Science and Technology, Lanzhou University, Lanzhou 730050, China; 7 Department of Plant Biology, Rutgers University, New Brunswick, NJ 08901, USA; jwhite3728@gmail.com

**Keywords:** *Achnatherum inebrians*, *Acyrthosiphon pisum*, growth and development, aqueous extract

## Abstract

**Simple Summary:**

Drunken horse grass *Achnatherum inebrians* is a widely distributed perennial poisonous grass on the grasslands of Northern and Northwestern China. The present study focused on contact toxicity of aqueous extracts of endophyte-infected (E+) and endophyte-free (E−) plants of *A. inebrians* in different growth periods on pea aphids. These results show that extracts from endophyte-containing plants may contain compounds that may be used to control insects and lay a foundation for deeper chemical analysis to identify insecticidal compounds.

**Abstract:**

The pea aphid *Acyrthosiphon pisum* has a worldwide distribution and causes serious losses for agricultural production. Drunken horse grass *Achnatherum inebrians* is a widely distributed perennial poisonous grass on the grasslands of Northern and Northwestern China. The present study focused on contact toxicity activity of aqueous extracts of endophyte-infected (E+) and endophyte-free (E−) *A. inebrians* in different growth periods of pea aphids, and the growth and development of two color morphs of F1 generation nymphs. Both of the color morphs had development durations in E+ treatments that tended to be longer at 1st, 2nd, 3rd, and 4th instars than E− and control (CK). The E+ treated aphids also showed decreased weights at maturity with over all lower mean relative growth rates (MRGR). Aphid survival of E+ treated aphids was lower than that of E− and CK at all growth periods. Seeding stage E+ extracts showed a greater propensity for negatively affecting aphids than did E+ extract at maturity and the yellowing stage. These results show that extracts from endophyte-containing plants may contain compounds that may be used to control insects.

## 1. Introduction

The pea aphid, *Acyrthosiphon pisum* (Hemiptera: Aphididae), has a worldwide distribution and causes serious losses for agricultural production. The pea aphid is mainly parthenogenetic, with short development periods and rapid reproduction. It is also known to be a highly destructive pests in agriculture [1], and is the major pest for many different kinds of leguminous plants [2]. Like other aphid species, it causes damage to host plants either directly by feeding on the phloem sap, and indirectly by transmitting plant viruses [3]. Since physical and biological controls are expensive, slow, inconsistent, and easily affected by climatic conditions, chemical control is widely used in China. However, over the long-term, it is not sustainable to use chemical pesticides because this results in development of resistance [4].

Drunken horse grass (*Achnatherum inebrians*) is a widespread perennial grass on alpine and subalpine grasslands of some provinces of China, including Inner Gansu, Xinjiang, Qinghai, Mongolia, and Tibet [5]. This grass is infected by a fungal endophyte *Epichloë gansuensis*. On the one hand, fungi and grasses form a mutually beneficial symbiosis, and the infected grasses are resistant to insects and drought and grow rapidly and competitively; On the other hand, the fungal endophyte produces toxic alkaloids, which cause great losses to grassland animal husbandry [6]. These endophytes produce alkaloids, including ergot alkaloids (e.g., ergometrine and ergonovine) and indole diterpenoids (e.g., lolitrem B). Ergot alkaloids have been shown to cause toxicity to mammals and insects [7].

The endophytic fungus of endophyte-infected *A. inebrians* (E+) leaves and seeds significantly reduces feeding by *Oedaleus* *decorus* and *Messor aciculatus* compared with endophyte-free *A. inebrians* (E−) [8]. A 72 h exposure to aqueous extracts of E+ *A. inebrians* reduced fecundity and prolonged population growth time in the pea aphid [9]. In other studies, involving different grass–endophyte associations, it was found that significantly more cereal leaf beetle larvae survived on uninfected plants than on infected tall fescue plants [10], whereas similar mortality rates were recorded on both uninfected plants and infected plants of *Phleum alpinum* accession from Russia [11]. The bird cherry oat aphid (*Rhopalosiphum padi*) preferred uninfected plants over *Epichloë*-infected plants of the wild grass Alpine timothy (*P. alpinum*) in choice experiments [12]. When rice leaf bug larvae (*Trigonotylus caelestialium*) feed on *Lolium multiflorum* that were artificially inoculated with *Neotyphodium uncinatum*, it was found that the larvae fed on endophyte-free plants were significantly larger than those fed on endophyte-infected plants. The survival rates of larvae were significantly higher on endophyte-free plants than endophyte-infected plants [13].

Pesticides can cause several disturbances to the environment, such as soil and groundwater pollution [14,15]. Safer alternatives are needed to control pests, such as the use of natural pesticides, which have been considered eco-friendly and easy to degrade products [16]. Insecticides may be developed to control herbivore and insect pathogens based on natural defensive chemicals of plants [17,18]. The deleterious effects of the phytochemicals or crude plant extracts on insects are manifested in several ways, such as toxicity [19], growth retardation [20], oviposition deterrence [21], reduction of fecundity and fertility [22]. The extracts from species of plant families, particularly Asteraceae, Maliaceae, Rutaceae and other families often exhibit entomotoxic properties [23].

Natural insecticides have played significant roles in pest management for decades, and extracts from endophyte containing plants may contain compounds that may be used to control insects. *A. inebrians* natural insecticidal constituents have not been previously explored. In this study, the pea aphid was used as the test insect in a contact toxicity activity experiment on the effects of aqueous extracts from E+ and E− plants of different growth periods. The aim of this study was to clarify the effects of aqueous extracts on the growth and development of two color morphs of pea aphids, and furthermore provide a theoretical basis for the effectiveness of drunken horse grass as aphid control agents in different growth periods.

## 2. Materials and Methods

### 2.1. Preparation of Aqueous Extracts from E+ and E− Plants

Twenty individual *A. inebrians* plants were harvested in Huangyuan county (Huangyuan site: N 36°26′, E 101°06′), Qinghai province in 2011. Fifteen tillers were selected to determine the presence or absence of the endophytic fungus *E. gansuensis* by microscopic (Carl Zeiss Suzhou Co., Ltd., Suzhou, Jiangsu, China) examination of leaf sheath pieces stained with 0.01% aniline blue (West Asia Chemical Technology Co., Ltd, Jinan, Shandong, China). The infection rate of plants was >95%. In 2012, the seeds of *A. inebrians* of similar sizes were collected from a plant. These seeds were divided into two parts. Prior to planting, one part was treated with thiophanate-methyl fungicide (Jiangsu dragon Light Chemical Co., Ltd.,Nanjing, Jiangsu, China) to eliminate the endophyte [24]. The seeds of both parts (198 seeds for each part) were separately planted in 18 field experiment plots (4.8 m × 4.0 m) on the Yuzhong Campus of Lanzhou University, China (Yuzhong site: N 35°87′, E 104°09′, elevation 1731 m) in 2013. The endophyte-infection status of all (392) plants were assessed using microscopic examination of leaf sheath pieces and seeds in 2013 and 2015. Based on the above results, these plants were denoted as E+ or E−. The grasses in seeding stage, maturity and yellowing stage were harvested in 2018, and the endophyte-infection status of all plants were again confirmed through microscopic examination using aniline blue stain. The results showed that endophyte infection was >95% for E+ and <5% for E− plants. Plants were dried in the shade, cut into sections, crushed and prepared for extraction.

To obtain aqueous extracts, the method of Shivendra [25] was changed slightly, where 100 g of grass samples were soaked with 400 mL of distilled water so that the aqueous extract concentration was 0.25 g/mL and was stirred every 12 h for 48 h in a 4 °C refrigerator (Shanghai aiyan Biotechnology Co., Ltd, Shanghai, China). The extract was filtered with three layers of medical gauze (Jinan Jianjia Medical Instrument Co., Ltd, Jinan, Shandong, China) 20 × 20 cm. Referring to the procedure of Liang [26], alkaloids were detected using an Agilent 1100 series high performance liquid chromatography (HPLC) system (Agilent Technologies, PaloAlto, CA, USA), ZORBAX-XDB C18 reversed phase chromatographic column (Agilent Technologies, PaloAlto, CA, USA), mobile phase flow rate of 1 mL/min, 20 ul and VWD ultraviolet detector (Agilent Technologies, PaloAlto, CA, USA).

### 2.2. Origin, Rearing and Preparation of the Pea Aphids

The pea aphids were originally obtained from an insect laboratory at Gansu Agricultural University, China in 2018. The pea aphids were reared on broad bean plants (*Vicia faba L*. variety Lin-can 5) and in growth chambers (Greenhouse Laboratory of Yuzhong Campus) under standard experimental conditions [relative humidity of 60 ± 10%, temperature of 23 ± 1 °C and 16:8 (light/dark) photoperiod]. After raising at least 20 generations, wingless adult aphids were used as the source of insects in these experiments.

### 2.3. Bioassay

Leaves with 10 adult pea aphids were immersed in aqueous extracts of E+ or E− plants, respectively, following the method of Shivendra [25]. After 5 s, the leaves were taken out, the excess liquid was quickly sucked off with filter paper, put into the Petri dish (diameter 9 cm) lined with moistened filter papers (Hangzhou Fuyang Beimu pulp and Paper Co., Ltd, Hangzhou, Zhejiang, China), and allowed to dry naturally, and then placed in an incubator (Zhejiang topyunnong Technology Co., Ltd, Hangzhou, Zhejiang, China) [relative humidity of 60 ± 10%, temperature of 23 ± 1 °C and 16:8 (light/dark) photoperiod]. Distilled water was employed as a control (CK). A total of 2700 adult (10 adult × 3 growth periods × 3 treatments × 30 replicates) were used with three growth periods (seeding stage, maturity, yellow stage), three treatments (E+, E−, CK), thirty replicates (30 replicates of different leaves) for pea aphid of green and red color morphs, respectively. The adult pea aphids on these leaves were considered the F_0_ population, and pea aphids were regarded to be dead when no movements were observed after being lightly touched on the hind legs [27,28,29]. For numbers of dead adult pea aphids were recorded after exposures for 24 h, 48 h and 72 h.

The nymphs born by pea aphids after exposure for 24 h were collected under each treatment and reared separately on fresh broad bean leaves (with the back of the blade facing up, every third day the bean blade was replaced) in 9 cm diameter Petri dish (Shanghai Xiyan Scientific Instrument Co., Ltd, Shanghai, China) lined with moistened filter papers [27]. The petiole was covered with moistened absorbent cotton (Qingdao Miaoren Medical Technology Co., Ltd, Qingdao, Shandong, China) to maintain moisture in leaves. One nymph was added to each Petri dish. A total of 2160 nymph (80 nymph × 3 growth periods × 3 treatments × 3 replicates) for of green and red color morphs, respectively. The growth conditions of nymphs were the same as above mentioned adults. Nymphs were considered the F_1_ population. Nymphs were weighed (W_1_), and every 12 h observations were made on survival, molting, slough skin times (i.e., one time molting is 1st instar, two times molting is 2nd instar, three times molting is 3rd instar, and four times molting is 4th instar.), until the nymphs became adult pea aphids, then aphids were weighed again (W_2_). Weight difference was calculated (WD = W_2_ − W_1_), development durations (The number of days from the first instar to mature, DD), mean relative growth rate [MRGR = (lnW_2_ − lnW_1_)/DD] [30,31], also, the numbers of surviving adults were recorded each day until death (longevity is the time from birth to death). In order to reduce the experimental error, a balance accurate to 1/100,000 g was used for weighing. For each weight, a single pea aphid was placed on filter paper, and the data were recorded.

### 2.4. Statistical Analysis

The present work were carried out as multiple two-factor experiments (randomization across different growth periods and endophyte treatment). And more, green colors morphs and red color morphs of pea aphids that separate analyses were run for each index caused by the effect of plant extracts from different growth periods (G: seeding stage, maturity, yellowing stage) and endophyte treatment (T: E+, E−, CK), and the growth periods and treatment interaction effects. Data were analyzed using the MIXED procedure of SPSS 21.0 (SPSS, Chicago, IL, USA) according to the following model: A = μ + G + T + (G × T) + ε, in which A is the dependent variable, μ is the overall mean, G is the effect of growth period, T is the effect of endophyte treatment, G × T is the interaction between growth period and endophyte treatment, and ε is the residual error. Plant extracts from different growth periods and endophyte treatment were fixed effects. When a significant effect was detected, the differences between means were assessed using Duncan (D) with a Bonferroni (B) correction of *p* = 0.05.

## 3. Results

### 3.1. Alkaloid Content

The alkaloid content in aqueous extract of *A. inebrians* at different growth periods (Table 1). E+ plants in the seeding stage contained 19.60 (mg/kg) ergometrine, E+ plants in maturity contained 12.80 (mg/kg) ergometrine, E+ plants in the yellowing stage contained 6.30 (mg/kg), and E− plant extracts contained no measurable ergometrine. Ergonovine was not detected in either E+ and E− plants.

### 3.2. Mortality

**Figure 1:**
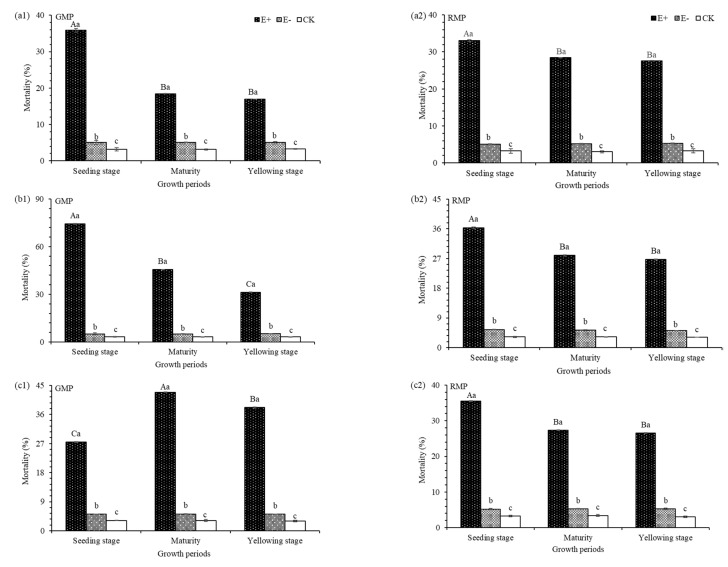
Mortality of green color morphs of pea aphids (GMP) and red color morphs of pea aphids (RMP) after treatment with aqueous extracts from plants after 24 h, 48 h and 72 h exposure (**a1**, **a2**, **b1**, **b2**, **c1** and **c2**). E+, endophyte-infected *A. inebrians*; E−, endophyte-free *A*. *inebrians*; CK, control. Different capital letters indicate that there are significant differences between different growth periods, and different small letters indicate that there are significant differences between different treatments (*p* < 0.05).

The green and red color morphs were significantly affected by E+ extract treatments at 24 h, 48 h, and 72 h (Table 2) when compared to controls. For the green color morphs of F_0_ pea aphids treated with E+ extract, the mortality rates at 24 h, 48 h and 72 h were 35.83%, 74.32% and 27.47%; for the red color morphs of F_0_ pea aphids treated with E+ extract the mortality rates at 24 h, 48 h and 72 h were 33.10%, 36.32% and 35.47%. Mortality was higher after exposure for 48 h compared to 24 h and 72 h. Aqueous extracts of E+ plants had significant mortality on F_0_ pea aphids compared to E− and CK (Figure 1).

### 3.3. The 1st Instar of F_1_ Pea Aphids

**Figure 2:**
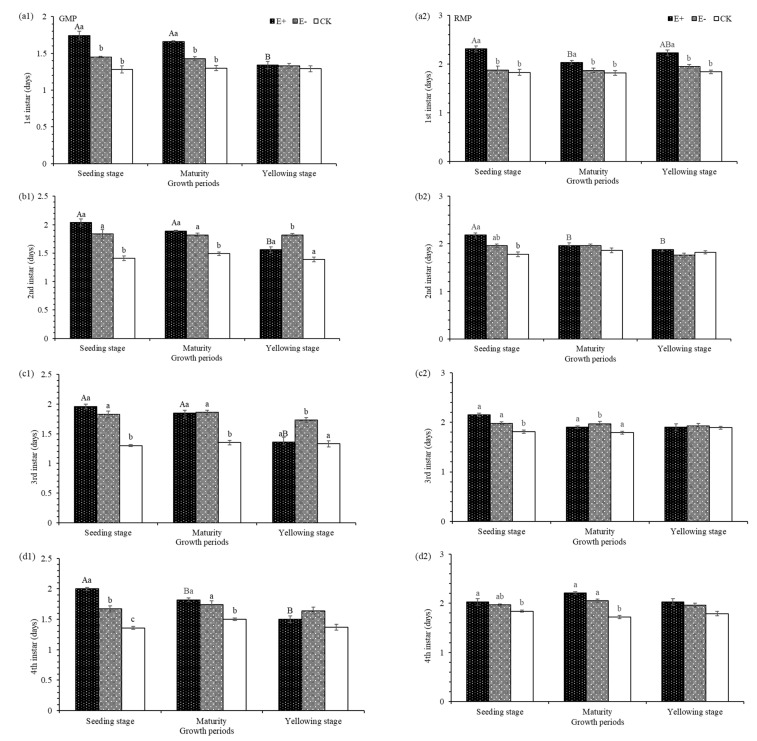
1st instar, 2nd instar, 3rd instar, and 4th instar growth times in green color morphs of pea aphid (GMP) and red color morphs of pea aphids (RMP) treated with aqueous extracts of different growth stages of plants (**a1**, **a2**, **b1**, **b2**, **c1**, **c2**, **d1** and **d2**). E+, endophyte-infected *A. inebrians*; E−, endophyte-free *A*. *inebrians*; CK, control. Different capital letters indicate that there are significant differences between different growth periods/stages, and different small letters indicate that there are significant differences between treatments (*p* < 0.05).

The 1st instar development times of the green and red color morphs were significantly affected by E+ plant treatments (Table 3) when compared to controls. The development time of the 1st instar green larvae was prolonged 0.49 d when treated with E+ seedling extracts but was less affected when treated with extracts from the mature and yellowing plant stages, which prolonged development by only 0.08 d and 0.40 d, respectively (Figure 2(a1). The 1st instar development time of the red color morphs of pea aphids was also significantly affected (Table 3). The development time of the 1st instar read larvae was prolonged 0.48 d when treated with extracts from E+ seeding stage, and slightly less affected (which prolonged development by 0.28 d and 0.08 d) when treated with extracts from the mature and yellowing stages of E+ plants, respectively (Figure 2(a2)).

### 3.4. The 2nd Instar of F_1_ Pea Aphids

The 2nd instar development times of the green and red color morphs were significantly affected by E+ treatments (Table 3) when compared to controls. The development time of the 2nd instar green larvae was prolonged 0.63 d when treated with E+ seedling extracts but was less affected when treated with extracts from the mature and yellowing plant stages, which were prolonged the 2nd instar by 0.40 d and 0.17 d, respectively (Figure 2(b1). The 2nd instar development time of red color morphs of pea aphids was also significantly affected (Table 3). The development time of the 2nd instar read larvae was prolonged 0.40 d when treated with extracts from E+ seedlings, and slightly less affected when treated with extracts from the mature and yellowing plant stages, which prolonged development by 0.1 d and 0.06 d, respectively (Figure 2(b2).

### 3.5. The 3rd Instar of the F_1_ Pea Aphids

The 3rd instar development times of the green and red color morphs were significantly affected by E+ extract treatments (Table 3) when compared to controls. The development time of the 3rd instar green larvae was prolonged 0.66 d when treated with E+ seedling extracts but was less affected when treated with extracts from the mature and yellowing plant stages, which were prolonged by 0.60 d and 0.03 d, respectively (Figure 2(c1)). The 3rd instar development time of red color morphs of pea aphids was also significantly affected (Table 3). The development time of the 3rd instar read larvae was prolonged 0.34 d when treated with extracts from E+ seeding stage, and slightly less affected when treated with extracts from the mature and yellowing plant stages, which prolonged development by 0.11 d and 0.01 d, respectively (Figure 2(c2)).

### 3.6. The 4th Instar of the F_1_ Pea Aphids

The 4th instar development times of the green and red color morphs were significantly affected by E+ extract treatments (Table 3) when compared to controls. The development time of the 4th instar green larvae was prolonged 0.64 d when treated with E+ seedling extracts but was less affected when treated with extracts from mature and yellowing plant stages, which were prolonged by 0.32 d and 0.13 d, respectively (Figure 2(d1)). The 4th instar development time of red color morphs of pea aphids was also significantly affected (Table 3). The development time of the 4th instar read larvae was prolonged 0.49 d when treated with extracts from mature E+ plants, and slightly less affected when treated with extracts from seedlings and yellowing E+ plants, which prolonged development by 0.19 d and 0.24 d, respectively (Figure 2(d2)).

### 3.7. The Biological Parameters of the F_1_ Pea Aphids

**Figure 3:**
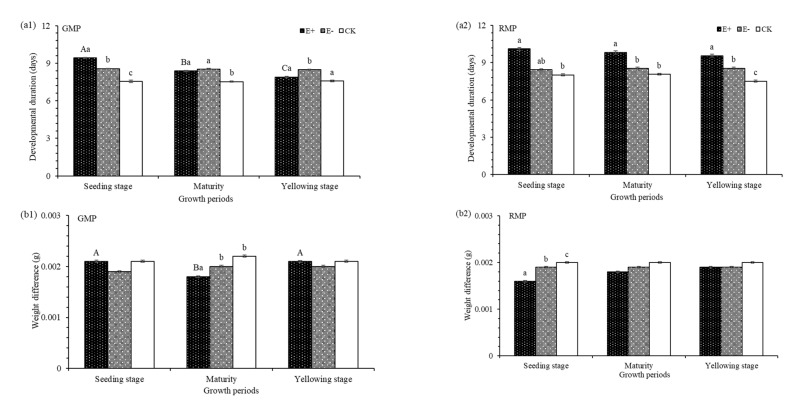

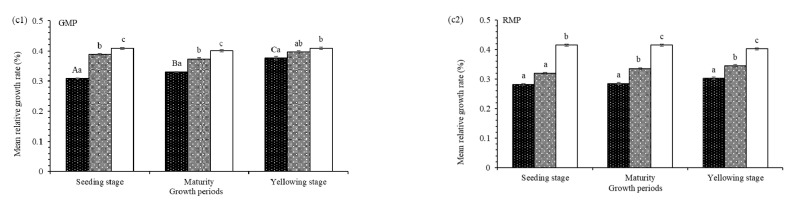
Developmental duration, weight difference and mean relative growth rate in green color morphs of pea aphids (GMP) and red color morphs of pea aphids (RMP) of *Achnatherum inebrians* with aqueous extracts of different growth periods (**a1**, **a2**, **b1**, **b2**, **c1** and **c2**). E+, endophyte-infected *A. inebrians*; E−, endophyte-free *A. inebrians*; CK, control. Different capital letters indicate that there are significant differences between different growth periods, and different small letters indicate that there are significant differences between different treatments (*p* < 0.05).

The development durations (DD), weight differences (WD) and mean relative growth rates (MRGR) of the green color morphs of pea aphids were significantly affected by E+ extracts taken from different plant growth periods and their interaction (Table 4). The longest DD was in aphids treated with extracts of E+ seedlings (9.43 d), which was 0.86 d and 1.88 d longer than E− and CK, respectively (Figure 3a1). The WD of E+ was the smallest (0.0018) in aphids treated with E+ extracts taken at plant maturity, which was 10% and 18% lower than that of E− and CK, respectively. The MRGR of aphids treated with extracts from E+ seeding stage was the lowest (0.3087), which was 20.52% and 24.52% lower than that of E− and CK, respectively (Figure 3(c1).

The DD and MRGR parameters of the red color morphs of pea aphids were also significantly affected by treatments, growth periods and their interactions (Table 4). The longest DD was seen in aphids treated with extracts from E+ seedlings; this was 10.11 d, which was 1.67 d and 2.09 d longer than E− and CK treated aphids, respectively (Figure 3(a2)). The WD was the smallest (0.0016) in aphids treated with extracts from E+ seeding stage, which was 15.79% and 20.00% lower than that of E− and CK, respectively (Figure 3(b2)). The MRGR of E+ was the lowest (0.2821) in aphids treated with extracts from E+ seeding stage, which was 11.93% and 31.99% less than that of E− and CK, respectively (Figure 3(c2)).

### 3.8. The Longevity and SP of the F_1_ Pea Aphids

**Figure 4:**
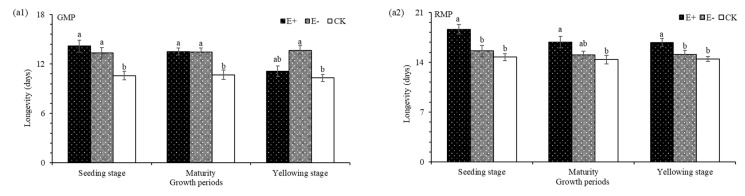
Longevity in green color morphs of pea aphids (GMP) and red color morphs of pea aphids (RMP) treated with aqueous extracts of plants in different growth periods/stages (**a1** and **a2**). E+, endophyte-infected *A. inebrians*; E−, endophyte-free *A. inebrians*; CK, control. Different capital letters indicate that there are significant differences between different growth periods, and different small letters indicate that there are significant differences between different treatments (*p* < 0.05).

The longevity of the green color morphs of pea aphids was significantly affected by E+ extract treatments (Table 5). The greatest longevity (14.18 d) was seen in the aphids treated with extracts from E+ seedlings, which was 0.86 d and 3.59 d longer than E− and CK plants, respectively (Figure 4(a1)). The longevity of red color morph of pea aphid was also significantly affected by E+ plant extract treatments (Table 5). The greatest longevity (18.62 d) was seen in the aphids treated with extracts from E+ seedlings, which was 3.04 d and 3.90 d longer than E− and CK treatments, respectively (Figure 4(b1)).

**Figure 5:**
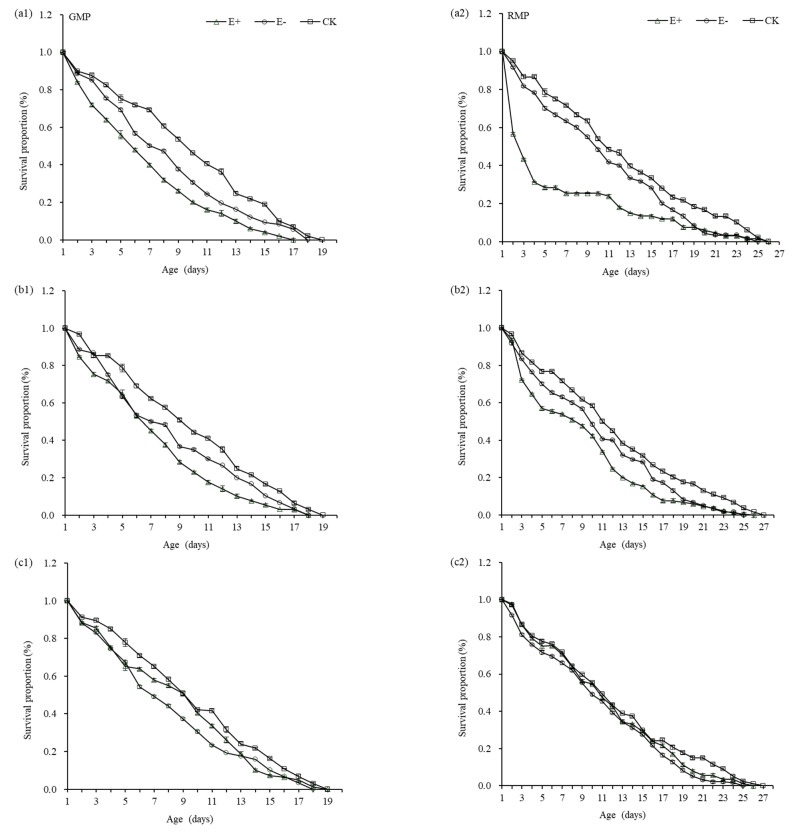
Survival proportion in green color morphs of pea aphids (GMP) and red color morphs of pea aphids (RMP) treated with aqueous extracts different growth periods(seeding stage, **a1**, **a2**; maturity, **b1**, **b2**; yelowing stage, **c1**, **c2**). E+, endophyte-infected *A. inebrians*; E−, endophyte-free *A. inebrians*; CK, control.

The survival curve of green pea aphids for extracts taken from different plant growth periods/stages are shown in Figure 5(a1–a3). The survival rate of the green pea aphids gradually decreased with the development stage, but there was no significant difference among treatments. The survival rate of nymphs decreased linearly under E+ treatment, and the survival rate was relatively low. The survival curves of red pea aphids at different growth periods were shown in Figure 5(b1–b3). The survival rates of red pea aphids gradually decreased with the development stage, and there were significant differences among treatments (Table 5). The nymph survival rate of aphids treated with extracts from E+ seedling decreased significantly from 0–4 d, and the 5–25 d survival rate decreased slowly; the survival rates of nymphs decreased linearly in maturity, and the survival rate was relatively low.

## 4. Discussion

Drunken horse grass (*Achnatherum inebrians*) is one of the main poisonous weeds on natural grasslands in North and Northwest China. It has strong survival ability and strong vitality. When the environmental conditions are suitable, it can reproduce rapidly. It has strong allelopathic effects on surrounding organisms, and has the characteristic of insect resistance [32], and infection by endophytic fungi of genus *Epichloë* significantly enhances insect resistance of drunken horse grass. Many investigators have shown that endophytic fungal symbionts of plants may increase plant resistance to insects, mites and nematodes [33,34]. Grass endophytic fungi have been shown to increase plant resistance to at least 45 species of Insecta [33,34]. Results in the literature show that the population density of *R. padi* and *Tetranychus cinnabarinus* on E+ plants is often significantly lower than that of E− plants [35]. In leaf feeding experiments results have shown that *T. cinnabarinus* prefers E− leaves compared to E+ leaves. Mortality of *R. padi* was 16.94% on the 1st day to 100% on the 7th day, and was significantly higher than that of aphids feeding on E− leaves [36]. This is consistent with our studies. Results in this paper show that the aqueous extracts of E+ plants at different growth periods/stages were found to have obvious toxicity effects on the mortality rates of adults and the growth of larvae of both of the color morphs of pea aphids.

In this study, the impacts of the aqueous extracts of *A. inebrians* were examined on adult pea aphids. The results showed that the toxicity of the aqueous extracts of E+ to green color morphs of F_0_ pea aphids caused 35.83%, 74.32% and 27.47%; and red color morphs of F_0_ pea aphids the mortality rates were 33.10%, 36.32% and 35.47%. Gouvêa Shaiene [37] showed that the toxicity of *Acmella oleracea* ethanol extracts to aphids *Myzus persicae* caused 90% mortality within 70 h and reduced their fecundity, whereas the aqueous extracts as control were inactive. Investigators prepared several sublethal doses of daphnetin by dissolving daphnetin in acetone solution, diluted to 0.05% and 0.025%, the mortality of *M. persicae* nicotianae was 35.67% and 4.09% after 12 h of contact, and 42.69% and 14.62% after 24 h contact [38]. After contact with pea aphids for 12 h, the mortality of pea aphids was 32.5% and 30.0%, respectively [39]. In the present study, the contact effects of E+ aqueous extracts on pea aphids were similar to the sublethal dose of commonly used insecticides. However, there was no significant effect between E− and CK for pea aphid. This present study identified only the E+ plant extracts as active in insecticidal effects. If its active components, perhaps fungus-produced alkaloids or other substances produced by plants in response to the fungus, were developed as a natural pesticide, the insecticidal effects could be enhanced. The E+ extract effects on mortality rates of pea aphids were higher in seedlings and generally decreased as plants aged. The reason may be that E+ seedlings contain endophytic fungi in high concentrations that are actively growing, and as plants mature and begin to senesce, fungi cease growth and metabolic activity [24].

To assess the influence of environmental factors on aphid growth and development, investigators generally use development durations, body weight differences and relative daily average body weight growth rates [40]. Daily average weight growth rate (MRGR) has been recognized as an ecological parameter reflecting insect variation [41,42]. It is simple and feasible to use these parameters to study the effects of extracts of E+ plants on the growth and development of aphids. In another study, *Plute xylostella* was treated with different sublethal concentrations of insecticides, the development duration of larvae was prolonged [43,44]. Similar to this, when a sublethal dose of imidacloprid was applied, the development duration and longevity of pea aphids were prolonged, and the survival rates were low [45]. In the present study, the instar growth times, weight differences, mean relative growth rates, longevity, and survival proportions after treatment with aqueous extracts of E+ plants showed significant negative effects. Among them, compared with the maturity and yellowing stage, E+ aqueous extracts required the longest time for all instars. DD and longevity of both morphs of pea aphids was lowest when treated with extracts from seedlings. Also, it was found that the green color morphs of pea aphids took the longest time for the 2nd instar and the shortest for the 1st instar. While the red color morphs of pea aphid took the longest time for the 1st instar and the shortest for the 4th instar, and the DD was longer than that the green color morphs of pea aphids. E+ plant extracts of seedlings prolonged the growth and development time of both color morphs of pea aphids and was not conducive to their growth and survival.

Extracts from endophyte-infected seedings were more potent compared with extracts from older plants. We hypothesize that toxicity of extracts to aphids is due to ergot alkaloids produced by the endophytic fungus. The difference between seedings and more mature plants may be due to higher concentrations of endophyte-produced ergot alkaloids in grass seedling tissues than in mature plant tissues [7]. Future work will need to be done to confirm the contact toxicity of the endophyte-produced alkaloids to aphids.

## 5. Conclusions

Drunken horse grass *Achnatherum inebrians* is a widely distributed perennial poisonous grass on the grasslands of Northern and Northwestern China. We assessed contact toxicity of aqueous extracts of endophyte-infected (E+) and endophyte-free (E−) plants of *A. inebrians* in different growth periods of pea aphids. We found that extracts from endophyte-containing seedlings were more potent than those from more mature plants. The endophytic fungus produces compounds that may be used to control insects as a biogenic insecticide. More study is needed to identify the specific insecticidal compounds.

## Figures and Tables

**Table 1:** Alkaloid content in aqueous extract of A. inebrians at different growth periods.

Alkaloids (mg/kg)	Seeding Stage		Maturity		Yellowing Stage	
	E+	E−	E+	E−	E+	E−
ergometrine	19.60	ND	12.80	ND	6.30	ND
ergonovine	ND	ND	ND	ND	ND	ND

Abbreviations: E+, endophyte-infected A. inebrians; E−, endophyte-free A. inebrians; ND, not detectable.

**Table 2:** Two-way ANOVA for the effects of growth periods (G) and treatments (T) on insecticidal activities of green color morphs of pea aphids (GMP) and red color morphs (RMP) of pea aphids.

Morphs	Factors	df (x, y) ^1^	24 h		48 h		72 h	
			F	*p*	F	*p*	F	*p*
GMP	G	2, 891	326.39	<0.001	4124.51	<0.001	739.96	<0.001
	T	2, 891	3212.62	<0.001	54931.30	<0.001	36638.42	<0.001
	G∗T	4, 891	340.69	<0.001	4127.97	<0.001	736.76	<0.001
RMP	G	2, 891	28.80	<0.001	123.16	<0.001	224.36	<0.001
	T	2, 891	6868.93	<0.001	12811.79	<0.001	18077.88	<0.001
	G∗T	4, 891	31.77	<0.001	121.66	<0.001	225.07	<0.001

^1^ x is numerator degree of freedom; y is the denominator degree of freedom. The same in the table below.

**Table 3:** Two-way ANOVA for the effects of growth periods (G) and treatments (T) on different instars of green color morphs of pea aphids (GMP) and red color morphs (RMP) of pea aphids.

Morphs	Factors	1st Instar			2nd Instar			3rd Instar			4th Instar		
		df (x, y)	F	*p*	df (x, y)	F	*p*	df (x, y)	F	*p*	df (x, y)	F	*p*
GMP	G	2, 544	7.07	0.001	2, 379	39.82	<0.001	2, 316	22.16	<0.001	2, 288	4.54	0.011
	T	2, 544	14.31	<0.002	2, 379	28.02	<0.001	2, 316	31.19	<0.001	2, 288	17.72	<0.001
	G∗T	4, 544	2.68	0.031	4, 379	10.46	<0.001	4, 316	6.51	<0.001	4, 288	2.37	0.053
RMP	G	2, 613	2.25	0.106	2, 459	14.78	<0.001	2, 404	8.81	<0.001	2, 354	0.46	0.630
	T	2, 613	25.55	<0.001	2, 459	12.21	<0.001	2, 404	5.14	0.006	2, 354	10.41	<0.001
	G∗T	4, 613	1.35	0.252	4, 459	1.740	0.140	4, 404	4.54	0.001	4, 354	4.18	0.003

**Table 4:** Two-way ANOVA for the effects of growth periods (G) and treatments (T) on the biological parameters of green color morphs of pea aphids (GMP) and red color morphs (RMP) of pea aphids.

Morphs	Factors	df (x, y)	Developmental Duration (DD)		Weight Difference (WD)		Mean Relative Growth Rate (MRGR)	
			F	*p*	F	*p*	F	*p*
GMP	G	2, 230	45.55	<0.001	5.98	0.003	82.45	<0.001
	T	2, 230	117.27	<0.001	3.69	0.026	41.40	<0.001
	G∗T	4, 230	24.30	<0.001	8.79	<0.001	6.31	<0.001
RMP	G	2, 318	15.34	<0.001	0.39	0.675	3.15	<0.044
	T	2, 318	181.36	<0.001	2.52	0.082	61.10	<0.001
	G∗T	4, 318	5.67	<0.001	0.46	0.768	2.78	0.027

**Table 5:** Two-way ANOVA for the effects of growth periods (G) and treatments (T) on the longevity and survival proportion (SP) of green color morphs of pea aphids (GMP) and red color morphs (RMP) of pea aphids.

Morphs	Factors	Longevity			Survival Proportion		
		df (x, y)	F	*p*	df (x, y)	F	*p*
GMP	G	2, 200	11.86	<0.001	2, 164	0.168	0.846
	T	2, 200	15.89	<0.001	2, 164	0.544	0.581
	G∗T	4, 200	2.41	0.051	4, 164	0.041	0.997
RMP	G	2, 299	1.13	0.326	2, 225	1.661	0.192
	T	2, 299	10.12	<0.001	2, 225	5.777	0.004
	G∗T	4, 299	0.261	0.903	4, 225	0.302	0.876

## Data Availability

All data contained within the article.

## References

[1] Sun Y.F., Qiao H.L., Ling Y., Yang S.X., Rui C.H., Pelosi P., Yang X.L. (2011). New analogues of (E)-β-farnesene with insecticidal activity and binding affinity to aphid odorant-binding proteins. J. Food Chem..

[2] Filomena D.B., Lea R., Daniele B., Annalisa G., Terenzio C., Yu F.S., Patrizia F. (2015). Expression pattern analysis of odorant-binding proteins in the pea aphid *Acyrthosiphon pisum*. Insect Sci..

[3] Du S.Q., Yang Z.K., Qin Y.G., Wang S.S., Duan H.X., Yang X.L. (2018). Computational investigation of the molecular conformation-dependent binding mode of (E)-β-farnesene analogs with a heterocycle to aphid odorant-binding proteins. J. Mol. Modeling.

[4] Wu J.X. (1999). Agricultural Entomology (Northern version). Ecological Basis of Pest Control.

[5] Shi Z.C. (1997). Important poisonous plants in Chinese Grassland, In *Study Toxicol*. Mech. Phytotoxin.

[6] Yao X., Chai Q., Chen T., Chen Z., Wei X., Bao G., Song M., Wei W., Zhang X., Li C. (2019). Disturbance by grazing and the presence of rodents facilitates the dominance of the unpalatable grass *Achnatherum inebrians* in alpine meadows of northern China. Rangel. J..

[7] Miles C.O., Lane G.A., diMenna M.E., Garthwaite I., Piper E.L., Ball O.J.P., Latch G.C.M., Allen J.M., Hunt M.B., Bush L.P. (1996). High levels of ergonovine and lysergic acid amide in toxic *Achnatherum inebrians* accompany infection by an *Acremonium*-like endophytic fungus. J. Agric. Food Chem..

[8] Zhang X.X., Li C.J., Nan Z.B. (2010). Effects of cadmium stress on growth and anti-oxidative systems in *Achnatherum inebrians* symbiotic with *Neotyphodium gansuense*. J. Hazard. Mater..

[9] Ma Y.L., Li C.J. (2021). Effect of the contact activity and population parameters of aqueous extracts of the *Achnatherum inebrians* against the pea aphid *Acyrthosiphon pisum*. Acta Ecol. Sin..

[10] Clement S.L., Bradley V.L., Elberson L.R., Bragg D.E., Phillips T.D. (2009). Cereal leaf beetle colonizes grass germplasm nurseries and impacts seed production activities. Forage Grazinglands.

[11] Clement S.L., Elberson L.R. (2010). Variable effects of grass-Neotyphodium associations on cereal leaf beetle (Coleoptera: Chrysomelidae) feeding, development and survival. J. Entomol. Sci..

[12] Clement S.L., Hu J.G., Stewart A.V., Wang B., Elberson L.R. (2011). Detrimental and neutral effects of a wild grass-fungal endophyte symbiotum on insect preference and performance. J. Insect Sci..

[13] Shiba T., Sasaki T., Kasai E. (2007). Resistance to the rice leaf bug (*Trigonotylus caelestialium*) is conferred by *Neotyphodium* endophyte infection of Italian ryegrass (*Lolium multiflorum*). Jpn. Soc. Grassl. Sci..

[14] Roditakis E., Vasakis E., Grispou M., Stavrakaki M., Nauen R., Gravouil M., Bassi A. (2015). First report of *Tuta absoluta* resistance to diamide insecticides. J. Pest Sci..

[15] Soares M.A., Campos M.R., Passos L.C., Carvalho G.A., Haro M.M., Lavoir A.V., Biondi A., Zappala L., Desneux N. (2019). Botanical insect cide and natural enemies: A potential combination for pest management against *Tuta absoluta*. J. Pest Sci..

[16] Mossa A.T.H. (2016). Green pesticides: Essential oils as biopesticides in insect-pest management. Environ. Sci. Technol..

[17] Regnault-Roger C., Vincent C., Arnasson J.T. (2012). Essential oils in insect control: Low-risk products in a high-stakes world. Annu. Rev. Entomol..

[18] Campos M.R., Biondi A., Adiga A., Guedes R.N.C., Desneux N. (2017). From the Western Palaearctic region to beyond: *Tuta absoluta* 10 years after invading Europe. Journal of Pest Science.

[19] Hiremath I.G., Ahn Y.J., Kim S.I. (1997). Insecticidal activity of Indian plant extracts against *Nilaparvata lugens* (Homoptera: Delphacidae). Appl. Entomol. Zool..

[20] Breuer M., Schmidt G.H. (1995). Influence of a short period treatment with *Melia azedarach* extract on food intake and growth of the larvae of *Spodoptera frugiperda* (J. E. Smith) (Lep., Noctuidae). J. Plant Dis. Prot..

[21] Dimock M.B., Renwick J.A.A. (1991). Oviposition by field populations of *Pieris rapae* (Lepidoptera: Pieridae) deterred by an extract of a wild crucifer. Environ. Entomol..

[22] El-Ibrashy M.T. (1974). Sterilization of the Egyptian cotton leafworm *Spodoptera littoralis* (Boisd.) with a foliage extract of *Podocarpus gracilior*. J. Appl. Entomol..

[23] Hermawan W., Kajiyama S., Tsukuda R., Fujisaki K., Koboyashi A., Nakasuji F. (1994). Antifeedant and antioviposition activities of the fraction of extract from a tropical plant, *Andrographis paniculata* (Acanthaceae), against the diamondback moth, *Plutella xylostella* (Lepidoptera:Yponomeutidae). Appl. Entomol. Zoolgy.

[24] Li C.J., Nan Z.B., Zhang C.J., Zhang C.Y., Zhang Y.H. (2009). Effects of drunken horse grass infected with endophyte on Chinese rabbit. J. Agric. Sci. Technol..

[25] Shivendra S. (2003). Effects of aqueous extract of neem seed kernel and azadirachtin on the fecundity, fertility and post-embryonic development. J. Appl. Entomol..

[26] Liang Y., Wang H.C., Li C.J., Nan Z.B., Li F.D. (2017). Effects of feeding drunken horse grass infected with *Epichloë gansuensis* endophyte on animal performance, clinical symptoms and physiological parameters in sheep. BMC Vet. Res..

[27] Biondi A., Desneux N., Siscaro G., Zappalà L. (2012). Using organic-certified rather than synthetic pesticides may not be safer for biological control agents: Selectivity and side effects of 14 pesticides on the predator *Orius laevigatus*. Chemosphere.

[28] Gou Y.P., Guo S.F., Wang G., Liu C.Z. (2020). Effects of short-term heat stress on the growth and development of *Bradysia cellarum* and *Bradysia impatiens*. J. Appl. Entomol..

[29] Zhao F., Xing K., Ary A.H., Ma C.S. (2019). The importance of timing of heat events for predicting the dynamics of aphid pest populations. Pest Manag. Sci..

[30] Chi B.J., Zhang X.H., Shi Q.H., Wang N.X., Liu L. (2018). Colored plastic films affect demographic characteristics of *Aphis gossypii* on cucumber plants. Int. J. Pest Manag..

[31] Hu Z.Q., Kang J.X., Zhao H.Y., Tang X.C., Hu X.S. (2010). Effect of UV-B radiation on biological characteristics of two body color of *Sitobion apenae* (Fab) offspring. Acta Ecol. Sin..

[32] Grace J.B., Anderson T.M., Ollf H., Scheiner S.M. (2010). On the specification of structural equation models for ecological systems. Ecol. Monogr..

[33] Li X.Z., Yao X., Li C.J., Nan Z.B. (2015). Potential analysis of grass endophytes *Neotyphodium* as biocontrol agents. Chin. J. Plant Ecol..

[34] Blankenship J.D., Spiering M.J., Wilkinson H.H., Fannin F.F., Bush L.P., Schardl C.L. (2001). Production of loline alkaloids by the grass endophyte, *Neotyphodium uncinatum*, in defined media. Phytochemistry.

[35] Li C.J., Nan Z.B., Volker H.P., Dapprich P.D., Liu Y. (2004). A new *Neotyphodium* species symbiotic with drunken horse grass (*Achnatherum inebrians*) in China. Mycotaxon.

[36] Zhang X.X., Li C.J., Nan Z.B., Matthew C. (2012). *Neotyphodium* endophyte increases *Achnatherum inebrians* (drunken horse grass) resistance to herbivores and seed predators. Weed Res..

[37] Gouvêa S.M., Carvalho G.A., Fidelis E.G., Ribeiro A.V., Farias E.S., Picanço M.C. (2019). Effects of paracress (*Acmella oleracea*) extracts on the aphids *Myzus persicae* and *Lipaphis erysimi* and two natural enemies. Ind. Crops Prod..

[38] Gao P., Liu S.G., Hou T.P., Hou R.T., Gao R. (2001). Study on biological activity of daphnetin against aphid. Acta Ecol. Sinca.

[39] Liu H.P., Zhang T.W., Liu C.Z., Yang L.Q. (2019). Toxicity of ochamaejasmin 1.6% EW and its mixuere with three kinds of bactericides against *Acyrthosiphon Pisum*. Mod. Agrochem..

[40] Thieme T., Heimbach U. (1996). Development and reproductive of cereal aphids (*Homoptera: Aphididae*) on winter wheat cultivals. IOBC/WPRS Bull..

[41] Zhong G.H., Hu M.Y., Weng Q.F., Ma A.Q., Xu W.S. (2001). Laboratory and field evaluations of extracts from *Rhododendron molle* flowers as insect growth regulator to imported cabbage worm, *Pieris rapae* L. (Lepidoptera:Pieridae). J. Appl. Entomol..

[42] Yu Q., Feng Y.T., Du E.Q., Guo X.J., Guo G.M., Zhang R.X. (2018). Effects of different sub-lethal concentrations of two pesticides on growth, development and fecundity of *Grapholita molesta* (Busck). Plant Prot..

[43] You L., Wang G.L., Tian S.G., Wei H.Y. (2013). Sublethal effects of four low-toxicity insecticides on the development and reproduction of the diamondback moth, *Plutella*
*xylostella* (Lepidoptera: Plutellidae). Acta Phytophylacica Sin..

[44] Quan L.F., Zhang H.J., Sun L., Li Y.Y., Yan W.T., Yue Q., Qiu G.S. (2016). Research advances in sublethal effect of pesticide. J. Agric..

[45] Wang X.Q., Liu C.Z., Qi F.P., Li Y.H. (2014). Effects of sublethal dosage of imidacloprid on the growth, development and population, parameter of two color morphs of pea aphid. Acta Agrestia Sin..

